# Predicting breast cancer risk in a racially diverse, community‐based sample of potentially high‐risk women

**DOI:** 10.1002/cam4.4721

**Published:** 2022-04-06

**Authors:** Rachel J. Meadows, Wilson Figueroa, Kate P. Shane‐Carson, Tasleem J. Padamsee

**Affiliations:** ^1^ Center for Epidemiology & Healthcare Delivery Research JPS Health Network Fort Worth Texas USA; ^2^ The Ohio State University Center for Health Outcomes and Policy Evaluation Studies, College of Public Health Columbus Ohio USA; ^3^ Division of Health Services Management & Policy College of Public Health, The Ohio State University Columbus Ohio USA; ^4^ Division of Human Genetics, Department of Internal Medicine Ohio State University Comprehensive Cancer Center Columbus Ohio USA

**Keywords:** breast cancer prevention, breast cancer risk, Claus model, Gail model, IBIS model, risk prediction, Tyrer‐Cuzick model

## Abstract

**Background:**

Identifying women with high risk of breast cancer is necessary to study high‐risk experiences and deliver risk‐management care. Risk prediction models estimate individuals' lifetime risk but have rarely been applied in community‐based settings among women not yet receiving specialized care. Therefore, we aimed: (1) to apply three breast cancer risk prediction models (i.e., Gail, Claus, and IBIS) to a racially diverse, community‐based sample of women, and (2) to assess risk prediction estimates using survey data.

**Methods:**

An online survey was administered to women who were determined by a screening instrument to have potentially high risk for breast cancer. Risk prediction models were applied using their self‐reported family and medical history information. Inclusion in the *high‐risk subsample* required ≥20% lifetime risk per ≥1 model. Descriptive statistics were used to compare the proportions of women identified as high risk by each model.

**Results:**

*N* = 1053 women were initially eligible and completed the survey. All women, except one, self‐reported the information necessary to run at least one model; 90% had sufficient information for >1 model. The *high‐risk subsample* included 717 women, of which 75% were identified by one model only; 96% were identified by IBIS, 3% by Claus, <1% by Gail. In the high‐risk subsample, 20% were identified by two models and 3% by all three models.

**Conclusions:**

Assessing breast cancer risk using self‐reported data in a community‐based sample was feasible. Different models identify substantially different groups of women who may be at high risk for breast cancer; use of multiple models may be beneficial for research and clinical care.

## Graphical abstract

Risk prediction models have rarely been applied to community‐based settings to identify women with high risk for breast cancer. This study applied three breast cancer risk prediction models to a racially diverse, community‐based sample. The results highlight differences between models in the identification of high‐risk women.

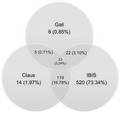

Lay Summary

Various risk prediction models incorporate different information to estimate an individual's lifetime risk of breast cancer. These models have mostly been used in clinical settings, omitting women who are not receiving healthcare. This study compared three different risk prediction models (Gail, Claus, and IBIS) among a community‐based sample to identify which women have high risk of getting breast cancer, using survey data collected directly from women. We found large differences in the number of women identified as high risk from each of the three models. We conclude that multiple models should be used to identify high‐risk women from the community.

## 1 BACKGROUND

In the United States, women with ≥20% lifetime risk of developing breast cancer are considered to be at high risk, compared to the 12% general population risk, and are recommended to receive risk‐management care.^1^, ^2^ Estimating breast cancer risk is essential for informing personal and clinical decision‐making for risk management. Women with high risk may seek additional information, genetic testing, and use clinically recommended risk‐management methods such as enhanced surveillance, prophylactic surgeries, and preventive medications.^3^ Enhanced surveillance methods can substantially improve early detection of breast cancer when treatment is most effective; prophylactic surgeries and medication can reduce breast cancer risk by 40%–95%.^1^, ^4^ Yet, these options are substantially underused due to lack of systematic risk screening and support in cancer risk‐management decision‐making in the United States.^3^, ^5^, ^6^, ^7^

Numerous breast cancer risk prediction models have been developed.^8^, ^9^ In the United States, the Gail, Claus, and IBIS (i.e., Tyrer‐Cuzick) models are commonly used in research and clinical practice. These models estimate lifetime risk of breast cancer up to age 80 years (Claus), 85 years (IBIS), or 90 years (Gail). The Gail model estimates risk of invasive breast cancer primarily based on nongenetic risk factors (e.g., race and medical/reproductive history), with limited familial history.^9^ The Claus model estimates risk of both invasive and noninvasive breast cancer based on first‐ and second‐degree familial history alone.^10^ The IBIS model estimates risk of both invasive and noninvasive breast cancer based on family history, pathogenic BRCA variants, and several indicators of endogenous estrogen exposure (i.e., menarche, parity, and hormone replacement therapy).^11^ Several reviews further explain differences between these risk prediction models.^8^, ^9^

Validation studies have observed substantial differences between the Gail, Claus, and IBIS models. The IBIS model may often provide the best predictive accuracy for breast cancer, while the Gail and Claus models more frequently underestimate breast cancer risk, particularly for women with strong family history of breast/ovarian cancer.^8^, ^9^ Yet, a recent study found that the Gail model had slightly higher discrimination compared to Claus and IBIS models in a U.S. mammography screening cohort of 35,921 women.^12^ Nonetheless, all three models over‐ or underestimate risk among certain subgroups (e.g., IBIS overestimates risk among women with atypical hyperplasia or lobular carcinoma in situ).^8^, ^13^ Each model confers advantages and disadvantages, and Gail, Claus, and IBIS models continue to be commonly used in research and clinical settings.^8^

The lack of systematic cancer risk assessment screening is a missed opportunity for both clinical care and research.^14^, ^15^ To date, most researchers have applied risk prediction models to medical record information, which may have high risk of selection bias and low internal validity due to excluding patients with missing data or models being calculated despite partial information.^16^, ^17^ Other approaches for identifying high‐risk women have been tested, such as surveys among unaffected family members of cancer registry patients, collecting information at the time of routine mammogram or in primary care settings, or from online questionnaires using public‐use websites.^15^, ^17^, ^18^, ^19^, ^20^ Few studies have assessed breast cancer risk using survey data collected directly from community‐based recruitment.^19^, ^20^ Expanding beyond clinic‐based settings offers a novel approach for identifying high‐risk women, which can facilitate research on high‐risk women's health‐related needs and aid in systematically directing women to appropriate clinical care.

Therefore, the goal of this study was to apply three breast cancer risk prediction models in a purposive racially diverse, community‐based sample of women using survey data. Our aims were (1) to investigate the proportion of women eligible to receive risk estimation by the Gail, Claus, and IBIS models and (2) to assess the resulting risk estimates.

## 2 METHODS

### 2.1 Recruitment and data collection

This study is part of the *Daughter Sister Mother Project*.^21^, ^22^, ^23^ Eligibility criteria included: (1) non‐Hispanic White or non‐Hispanic Black or African American racial‐ethnic identification, (2) age between 18 and 75 years, (3) no personal history of cancer (except nonmelanoma skin cancer), and (4) were determined as potentially having high risk for breast cancer via the online eligibility screener. Participants were recruited from (1) online databases of research volunteers (i.e., ResearchMatch and Study Search) (2) Facing Our Risk of Cancer Empowered (FORCE; a national nonprofit serving individuals with high risk of hereditary cancer), (3) Facebook, and (4) genetics and high‐risk breast programs at the Ohio State University Comprehensive Cancer Center and other U.S. clinical centers.

Recruitment materials advertised the study for white and African American women 18–75 years old, with a family history of breast cancer or a BRCA mutation, who had never had cancer themselves. Interested individuals completed an online screening instrument designed to distinguish women with potentially high risk for breast cancer. The screener asked up to 12 questions about race, ethnicity, age, family breast cancer history, and personal medical and genetic testing history. Individuals who passed through the screener were deemed eligible to continue and were invited to provide electronic informed consent and then participate in the online study survey. Surveys were administered October 2018–August 2019 using the REDCap data collection system, available on any Internet‐enabled device. Participants took an average of 31 min to complete the survey and received a $10 amazon.com gift card after completing the survey (potential participants who completed the screener only did not receive the incentive). Given our online data collection format, we then reviewed each completed survey to identify and remove fraudulent surveys. Fraudulent surveys, resulting from individuals completing the survey repeatedly or deploying programmed bots to harvest incentive payments, are a common problem in online studies.^24^ Our methods for identifying fraudulent surveys included screening for completion times too short for a human being to achieve, impossible ages, inconsistent information on repeated questions, false confirmation phone numbers, and more; these methods are detailed separately.^25^ This research was approved by the Ohio State University Institutional Review Board (Protocol #2017C0212) prior to study commencement.

### 2.2 Measures

#### 2.2.1 Known pathogenic variants in cancer predisposing genes

Participants were asked about history of genetic testing and the result, if applicable (i.e., negative, positive for *BRCA1*, *BRCA2*, or other pathogenic variants).

#### 2.2.2 Family history

Participants reported breast (unilateral and bilateral) and ovarian cancers and ages of diagnoses for their mother, sisters, daughters, aunts, grandmothers, half‐sisters, female first cousins, nieces, father, and brothers.

#### 2.2.3 Demographic characteristics

Sociodemographic information was collected using standardized questions about age, race, education, and annual household income.

#### 2.2.4 Additional measures

##### Specialist care

A dichotomous variable was created to indicate whether participants had ever seen a breast, cancer, or genetics specialist.

##### Perceived risk of breast cancer

Participants answered the following question: “What do you think your chance is of developing breast cancer in your lifetime?”. Responses were categorical ranging from 0% to 100% in 20% increments.

### 2.3 Data analysis

#### 2.3.1 Application of risk prediction models

We applied the Gail (original version), Claus (original version), and IBIS (version 7) risk prediction models to estimate each participant's lifetime risk of breast cancer.^9^, ^10^, ^11^ Models were run manually by research assistants trained by the third author (KSC), an experienced genetic counselor and researcher. For each participant, we ran only the models that could appropriately be utilized according to the model's eligibility criteria; this replicates the method that genetics clinics use in assessing breast cancer risk. For example, the Gail model is only suitable for women 35 years or older without reported pathogenic BRCA variants; the Claus model is only applicable to women who report first‐ or second‐degree relatives with breast cancer; the IBIS model has no eligibility restrictions.

#### 2.3.2 Categorization of participants into subsamples

Participants were categorized into one of two subsamples based on self‐reported BRCA status and risk prediction estimates.

##### High‐risk subsample

Includes participants with ≥20% lifetime breast cancer risk according to ≥1 risk prediction model or self‐report of a pathogenic BRCA1/2 variant.

##### Average‐risk subsample

Includes participants that the screener determined as potentially having high risk of breast cancer, who completed the survey, but lifetime risk estimates were <20% according to all three risk estimation models.

#### 2.3.3 Descriptive analyses

Descriptive statistics were conducted to compare demographic characteristics of our high‐risk (*n* = 717) and average‐risk (*n* = 336) subsamples. We also calculated the number of models applied each participant and cataloged reasons that models could not be run.

#### 2.3.4 Risk prediction estimates

Within the high‐risk subsample, we calculated percentages of women identified as high risk by each model. We conducted a quality control check by re‐estimating all models for a random 5% sample (*n* = 36) of the high‐risk subsample. We report median and ranges of risk scores calculated by each model, stratified by BRCA status. Discrepancies in risk scores were calculated as follows: (1) if all three models could be run, discrepancy = absolute difference between largest and smallest risk scores, (2) if only two models could be run, discrepancy = absolute difference between the two risk scores, (3) if only one model could be run, discrepancy was not calculated.

In supplemental analyses, we investigated differences in risk estimates by recruitment source, sociodemographic factors, perceived risk of breast cancer, and specialist use. All analyses were conducted using STATA version 16.^26^ Additional details about recruitment, variables, and supplemental analyses are in the Supplemental File.

## 3 RESULTS

### 3.1 Descriptive characteristics

Of the 1053 women who completed the survey, 717 (68%) were included in the high‐risk subsample. Compared to the high‐risk subsample, members of the average‐risk subsample (*n* = 336) were more likely to be ≥55 years old, Black or African American (even after adjustment for income and education), had lower household income, education, perceived breast cancer risk, and were less likely to have seen a cancer, breast, or genetics specialist (*p* < 0.05).

The high‐risk subsample was comprised of 65% White women and 35% Black or African American women. Most (95%) were recruited from nonclinical sources and 54% had never seen a specialist. The majority (65%) were 25–50 years old, had at least a college degree (74%), and reported a family history of at least one first‐degree relative with breast cancer (59%). Twenty percent reported having BRCA1/2 pathogenic variants (Table 1).

**TABLE 1:** Sample characteristics (*n* = 1053)

	High‐risk subsample (*n* = 717)	Average‐risk subsample (*n* = 336)	Missing (%)
	*n* (%)	*n* (%)	
Race/ethnicity			0%
Non‐Hispanic White	466 (65%)	155 (46%)	
Non‐Hispanic Black or African American	251 (35%)	181 (54%)	
Age			<1%
18–24 years	91 (13%)	24 (7%)	
25–34 years	228 (32%)	62 (19%)	
35–50 years	238 (33%)	81 (24%)	
1–65 years	141 (20%)	55 (16%)	
66–74 years	19 (3%)	113 (34%)	
Pathogenic variants			<1%
Not tested	458 (64%)	280 (83%)	
Negative	76 (11%)	37 (11%)	
*BRCA1, BRCA2*, or both	146 (20%)	0	
Other mutation	20 (3%)	0	
Missing	11 (2%)	19 (6%)	
Marital status			3%
Single	248 (35%)	96 (29%)	
Married/domestic partnership	367 (51%)	156 (46%)	
Divorced/separated/widowed	75 (11%)	74 (22%)	
Perceived breast cancer risk			<1%
0%	16 (2%)	20 (6%)	
1%–19%	131 (18%)	89 (26%)	
20%–59%	402 (56%)	201 (59%)	
60%–99%	163 (23%)	24 (7%)	
100%	5 (<1%)	1 (<1%)	
First‐degree relative(s) with breast cancer	423 (59%)	88 (26%)	0%
Education			<1%
≤High school	31 (4%)	98 (29%)	
Some college/tech school	152 (21%)	119 (35%)	
College graduate	271 (38%)	96 (29%)	
Postgraduate	262 (37%)	21 (6%)	
Annual household income			8.9%
<$50,000	189 (26%)	121 (36%)	
$50,000–$99,999	220 (31%)	111 (33%)	
$100–$199,999	191 (27%)	62 (19%)	
≥$200,000	52 (7%)	13 (4%)	
Ever seen a cancer or genetics specialist	332 (46%)	81 (24%)	<1%
Recruitment source			1%
ResearchMatch/Study Search	372 (52%)	232 (69%)	
FORCE	116 (16%)	4 (1%)	
Healthcare provider	34 (5%)	13 (4%)	
Facebook	60 (8%)	39 (12%)	
Advertisement, referral, other	127 (18%)	43 (13%)	

### 3.2 Number of risk models run

**FIGURE 1:**
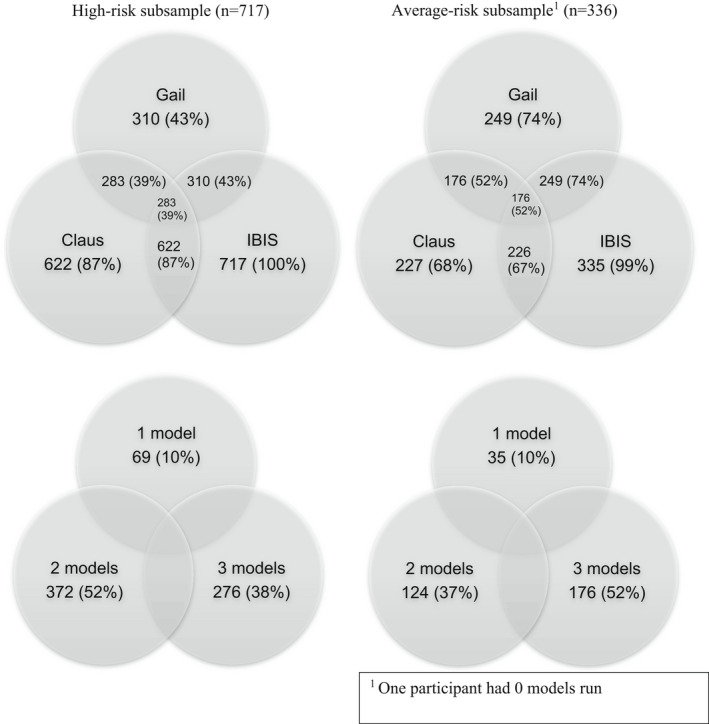
Number of participants eligible for application of each risk prediction model

The Gail model was run for 53% of our total sample; Claus was run on 81%, and IBIS was run on 99.9%. Only one participant did not provide sufficient information to run any model. Ten percent of participants had only one model run (all IBIS), 47% had two models run (9% Gail and IBIS, 38% Claus and IBIS), and 43% had all three models run (Figure 1). Figure 1 presents the number and proportions of participants eligible for each risk prediction model, stratified by subsample. The reasons for models not being applied are presented in Table 2.

**TABLE 2:** Reasons and frequencies of models not run, stratified by subsample

	High‐risk subsample	Average‐risk subsample
Gail	<35 years of age (*n* = 319, 67%) ≥35 years of age, but reported pathogenic BRCA mutation (*n* = 88, 18%)	<35 years of age (*n* = 86, 99%) Missing participant's age (*n* = 1, <1%)
Claus	No affected 1st or 2nd degree relatives (*n* = 32, 7%) Missing age of diagnosis for all affected family members (*n* = 37, 8%) Missing ages for some affected family members (*n* = 25, 100%) Implausible age of diagnosis (*n* = 1, <1%)	Missing age of diagnosis for all affected family members (*n* = 42, 39%) No affected relatives (*n* = 51, 47%) No affected 1st or 2nd degree relatives (*n* = 16, 15%)
IBIS	N/A	Missing participant's age (*n* = 1, <1%)

Supplemental analyses indicated positive associations between age and (1) having seen a specialist, and (2) having three models run compared to 1–2 models run. Participants recruited from Facebook had 2.4 times higher odds of having three models run compared to other recruitment sources. No differences were observed in the number of models run across race, income, education, or perceived risk of breast cancer (Table S1).

### 3.3 Findings relevant to the high‐risk subsample

#### 3.3.1 Proportion of high‐risk women identified by each model

**FIGURE 2:**
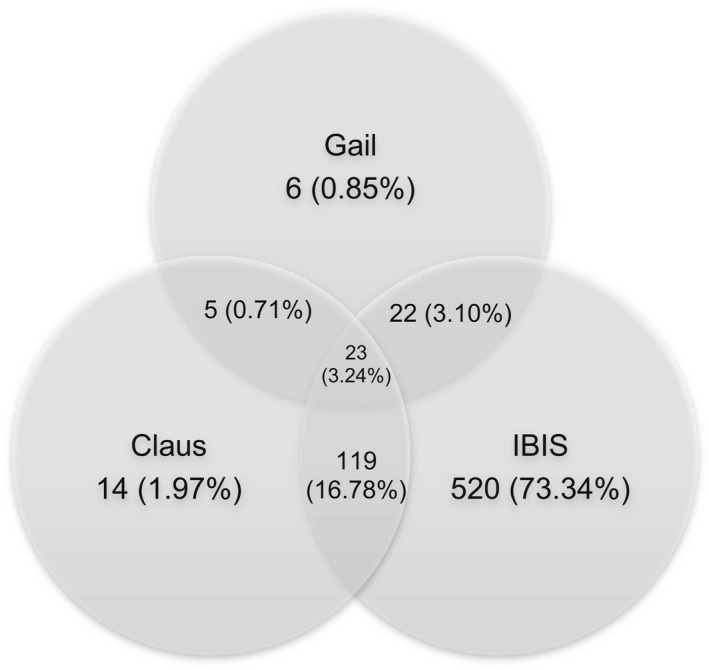
High‐risk subsample that met 20% risk threshold (*n* = 709). *Note:* 8 participants reported pathogenic BRCA variants and therefore were included in the high‐risk subsample (resulting in a total *n* = 717), despite not meeting the 20% risk threshold on Gail, Claus, or IBIS models

The high‐risk subsample included 709 women who had ≥20% lifetime risk from ≥1 model and eight women whose risk scores were <20% but self‐reported pathogenic BRCA variants. Of the 709 women, 76% had ≥20% risk according to only one model (73% IBIS, 2% Claus, and 1% Gail), 20% had ≥20% risk according to two models, and 3% had ≥20% risk according to all three models (Figure 2). The 5% quality control check resulted in no changes in eligibility for the high‐risk subsample, and the few differences in predicted risk scores were <5%.

#### 3.3.2 Differences in risk estimates by demographic & genetic factors

Estimated lifetime risks were 32.5% higher, on average, among women with reported pathogenic BRCA variants compared to women without (*p* < 0.001). Table 3 presents the median and range of risk values calculated by the Claus, Gail, and IBIS models, stratified by BRCA status. The highest risk percentages, up to 89.6%, were estimated by the IBIS model only (i.e., the only model to incorporate BRCA status). Nevertheless, the IBIS model calculated higher risk for both women with pathogenic BRCA variants and women without. Supplemental analyses revealed slightly higher risk scores for those who had more education, saw a specialist, or were recruited from FORCE or a healthcare provider (Table S2).

**TABLE 3:** Lifetime risk prediction estimates for the high‐risk subsample

Risk model	Min	Max	Median	Total *n* ^a^
Pathogenic BRCA variant carriers				
Gail	N/A^b^			0
Claus	8.3%	43.7%	16.5%	113
IBIS	15.0%	89.6%	63.3%	146
Women without known pathogenic BRCA variants				
Gail	6.6%	49.6%	15.0%	302
Claus	8.3%	43.7%	13.2%	509
IBIS	7.0%	64%	25.6%	571

^a^ Total does not equal 717 because participants could have >1 model run.

^b^ The Gail model was not run on women with pathogenic BRCA variants.

#### 3.3.3 Discrepancies between risk models

Among the high‐risk subsample, 52% had two models run and 38% had all three models run. Discrepancies between risk estimates were larger among women with pathogenic BRCA variants compared to women without pathogenic BRCA variants. Among women with two models run, the median discrepancy was 45.8% (range: 0.5%–74.7%) for those with pathogenic BRCA variants and 15.5% (range: 0.1%–74.7%) for those without pathogenic BRCA variants. For women who had all three models run (none of whom reported BRCA mutations), the median discrepancy was 14.3% (range: 1.4%–32.7%) (Table 4). Supplemental analyses also found smaller discrepancies associated with increasing age and among pathogenic BRCA variant carriers who are Black or African American or had never seen a specialist (Table S3).

**TABLE 4:** Discrepancies between lifetime risk estimates within the high‐risk subsample

Discrepancies for those with two models run (*n* = 372)							
No known pathogenic BRCA variant (*n* = 259)				Pathogenic BRCA variant carriers (*n* = 113)			
Min	Max	Median	Mean	Min	Max	Median	Mean
0.1%	48.2%	14.1%	14.3%	0.5%	74.7%	45.8%	40.5%

Discrepancies for those with three models run (*n* = 276)							
No known pathogenic BRCA variant (*n* = 276)				Pathogenic BRCA variant carriers (*n* = 0)			
Min	Max	Median	Mean	Min	Max	Median	Mean
1.4%	32.7%	14.3%	14.8%	N/A^a^			

^a^ Women with BRCA pathogenic variants did not have the Gail model run, therefore could have a maximum of two models run.

## 4 DISCUSSION

In our racially diverse, community‐based sample of 1053 women, the Gail, Claus, and IBIS models identified substantially different proportions of women as having ≥20% lifetime risk for breast cancer. The IBIS model identified the highest proportion of women as high risk, followed by Claus, then Gail. Only 3.2% of women were identified as high risk by all three models. Our community‐based research sample provides useful insights for identifying high‐risk women outside of clinical care settings that can inform both research and healthcare delivery.

Prior studies have most often applied risk prediction models to identify high‐risk women using electronic medical record (EMR) data and have reported similar differences in risk estimate patterns between models. Several large studies among mammogram patients have shown that the Gail model under‐identified potentially high‐risk women compared to IBIS, Claus, and other criteria such as the U.S. Preventive Service Task Force (USPSTF) family history criteria.^16^, ^17^, ^18^ Similar to the present study, Ozanne and colleagues^17^ reported that the IBIS model identified the highest proportion of the study population as high risk (5.6% identified by IBIS compared to 0.9% by Claus). Our study adds to the existing evidence that various risk models will identify substantially different proportions of potentially high‐risk women. Furthermore, the use of a single risk model is likely insufficient, and the use of multiple models captures a higher proportion of potentially high‐risk women.

Recently, alternative screening methods have been tested to identify potentially high‐risk women who should be referred to genetic counseling or testing within primary care. These methods (e.g., Referral Screening Tool, FHQ‐7, N‐TRAC) do not provide lifetime risk estimates but are more time‐efficient and provide a threshold to indicate the need for a clinical referral.^2^, ^8^ Objective cancer risk estimates among community‐based samples may facilitate more in‐depth research on personal risk perception and impacts on decision‐making processes, particularly among high‐risk women that are not engaged in clinical care.

The advantages of our community‐based approach to identifying high‐risk women compliment those of other approaches that also attempt to overcome the limitations of clinic‐based identification strategies. Contacting at‐risk family members through state cancer registries is one step in the right direction toward community‐based identification of high‐risk women, but relying on registry patients to respond to surveys and offer contact information for willing family members limits the potential to identify all women who may be at high risk. For example, in one study, 33% of cancer survivors responded to the initial survey request, and 52% of their at‐risk relatives subsequently responded.^15^ Recruiting women directly from community sources may facilitate a broader reach to additional groups of potentially high‐risk women. Overall, a focus on community‐based recruitment using survey data may help to better identify high‐risk women who have not yet entered risk‐related clinical care and facilitate research on risk‐related experiences and needs of the full population of high‐risk women.

Among the high‐risk subsample in this study, discrepancies between risk models were larger among women with pathogenic BRCA variants compared to women without pathogenic BRCA variants. This pattern could be explained by several potential mechanisms. Pathogenic BRCA variant carriers can have lifetime risks from 41% to 90%, and this may be a stronger driver of risk discrepancies than other risk factors.^1^ In addition, the Claus model does not take BRCA status into account (therefore predicting lower risk) while IBIS does (predicting a higher risk). Although women without known pathogenic BRCA variants had smaller discrepancies, the largest discrepancy among noncarriers was still 48.5%. Large discrepancies represent medical uncertainties, which may cause anxiety or confusion about perceived breast cancer risk; these emotions are among the most influential drivers of screening and prevention behavior.^3^ Risk uncertainty may be particularly consequential for high‐risk women without known pathogenic variants because they may rely on risk estimates to help guide risk‐management decision‐making more heavily than pathogenic variant carriers who have specific guideline‐recommended courses of preventive action available.

Several limitations are relevant to this study. Our data were entirely self‐reported, therefore pathogenic BRCA mutations and medical history were not objectively verified. Furthermore, family history data collected using surveys may be more susceptible to errors than family history information collected via clinician–patient discussion.^18^ Our study also included only two major U.S. racial groups, non‐Hispanic White, and non‐Hispanic Black or African American, to provide substantive analyses of differences between these two racial groups within the limitations of our data collection resources. Better understanding and effective strategies are critically needed to help overcome notable racial disparities documented in use of clinical risk‐ management services for hereditary breast and ovarian cancer between Black and White women^.^ ^3^, ^27^, ^28^, ^29^ Future studies would benefit from including sizeable subsamples of the other major U.S. racial‐ethnic groups as well (Asian/Asian American, Native American/Alaska Native, Hispanic/Latine). Lastly, half our participants were recruited from ResearchMatch, which comprises women with higher education and income levels, therefore our results may have limited generalizability to other racial/ethnic groups or women with lower socioeconomic status. Nonetheless, a strength of our study is that we recruited a community‐based sample of potentially high‐risk women, of whom <40% had ever seen a cancer or genetics specialist for high‐risk care. Because most high‐risk studies recruited from clinical settings and therefore include women who have already accessed care^8^, our study sheds light on the application of risk prediction models among women who have not yet accessed specialized high‐risk care.

Several opportunities exist to improve identification of high‐risk women to facilitate better linkage to clinical care and increase capacity for research.^30^ Accuracy of risk prediction models could be improved by incorporating factors that impact breast cancer risk, such as lifestyle behaviors, and by developing and validating models among racial/ethnic and socioeconomically diverse women.^31^ Improved accuracy among subgroups of women may also help reduce discrepancies between models and minimize medical uncertainty. Within the clinical context, EMRs could provide the basis for reliable, systematic risk screening in primary care settings if improvements in data collection were implemented to minimize missing or incomplete data.^32^ IT‐enabled automation of risk prediction models, in both clinical and community‐based settings, will help support feasibility and sustainability of future research.^33^

## 5 CONCLUSION

Our study demonstrated success in applying risk prediction models using survey data to identify high‐risk women through community‐based recruitment. The risk prediction results also demonstrated that the selection of risk prediction models can make a substantial difference in the identification of high‐risk women. Relying on any single model as a basis for screening or identification of high‐risk women is likely to miss a high proportion of potentially eligible women. Concurrent use of multiple models is likely most advisable.

## CONFLICT OF INTEREST

All authors have no conflict of interest to disclose.

## AUTHOR CONTRIBUTIONS

Rachel J. Meadows: Conceptualization, data analyses, writing, and editing. Wilson Figueroa: Conceptualization, writing, and reviewing. Kate P. Shane‐Carson: Conceptualization, data analyses, and reviewing. Tasleem J. Padamsee: Funding acquisition, conceptualization, writing, reviewing, and editing.

## ETHICS APPROVAL AND CONSENT TO PARTICIPATE

This research was approved by the Ohio State University Institutional Review Board (Protocol #2017C0212) prior to study commencement. Informed consent was obtained electronically from all research participants.

## PRECIS

Risk prediction models have rarely been applied to community‐based settings to identify women with high risk for breast cancer. This study applied three breast cancer risk prediction models to a racially diverse, community‐based sample and the results highlight differences between models in the identification of high‐risk women.

## Supporting information


**Appendix** S1

## Data Availability

Data sharing is not applicable to this article as no new data were created or analyzed in this study.

## References

[1] Daly MB , Pal T , Berry MP , et al. Genetic/familial high‐risk assessment: breast, ovarian, and pancreatic, version 2.2021, NCCN clinical practice guidelines in oncology. J Natl Compr Cancer Netw. 2021;19(1):77‐102. doi:10.6004/jnccn.2021.0001 33406487

[2] US Preventive Services Task Force , Owens DK , Davidson KW , et al. Risk assessment, genetic counseling, and genetic testing for BRCA‐related cancer: US preventive services task Force recommendation statement. JAMA. 2019;322(7):652‐665. doi:10.1001/jama.2019.10987 31429903

[3] Padamsee TJ , Wills CE , Yee LD , Paskett ED . Decision making for breast cancer prevention among women at elevated risk. Breast Cancer Res. 2017;19(1):34. doi:10.1186/s13058-017-0826-5 28340626 PMC5366153

[4] Ludwig KK , Neuner J , Butler A , Geurts JL , Kong AL . Risk reduction and survival benefit of prophylactic surgery in BRCA mutation carriers, a systematic review. Am J Surg. 2016;212(4):660‐669. doi:10.1016/j.amjsurg.2016.06.010 27649974

[5] Meadows RJ , Padamsee TJ . Financial constraints on genetic counseling and further risk‐management decisions among U.S. women at elevated breast cancer risk. J Genet Couns. Published online March 21, 2021;30(5):1452–1467. doi:10.1002/jgc4.1413 33749063 PMC12505379

[6] Padamsee TJ , Hils M , Muraveva A . Understanding low chemoprevention uptake by women at high risk of breast cancer: findings from a qualitative inductive study of women's risk‐reduction experiences. BMC Womens Health. 2021;21(1):157. doi:10.1186/s12905-021-01279-4 33863327 PMC8052843

[7] Padamsee TJ , Meadows R , Hils M . Layers of information: interacting constraints on breast cancer risk‐management by high‐risk African American women. Ethn Health Published Online December 27, 26 2018:1–24. doi:10.1080/13557858.2018.1562053 PMC9529154 30589360

[8] Cintolo‐Gonzalez JA , Braun D , Blackford AL , et al. Breast cancer risk models: a comprehensive overview of existing models, validation, and clinical applications. Breast Cancer Res Treat. 2017;164(2):263‐284. doi:10.1007/s10549-017-4247-z 28444533

[9] Gail MH . Twenty‐five years of breast cancer risk models and their applications J Natl Cancer Inst 2015;107(5):djv042. doi:10.1093/jnci/djv042 25722355 PMC4651108

[10] Claus EB , Risch N , Thompson WD . Autosomal dominant inheritance of early‐onset breast cancer. Implications for risk prediction. Cancer. 1994;73(3):643‐651. doi:10.1002/1097-0142(19940201)73:3<643::aid-cncr2820730323>3.0.co;2-5 8299086

[11] Tyrer J , Duffy SW , Cuzick J . A breast cancer prediction model incorporating familial and personal risk factors. Stat Med. 2004;23(7):1111‐1130. doi:10.1002/sim.1668 15057881

[12] McCarthy AM , Guan Z , Welch M , et al. Performance of breast cancer risk‐assessment models in a large mammography cohort. J Natl Cancer Inst. 2020;112(5):489‐497. doi:10.1093/jnci/djz177 31556450 PMC7225681

[13] Valero MG , Zabor EC , Park A , et al. The Tyrer‐Cuzick model inaccurately predicts invasive breast cancer risk in women with LCIS. Ann Surg Oncol. 2020;27(3):736‐740. doi:10.1245/s10434-019-07814-w 31559544 PMC7500748

[14] Evans O , Manchanda R . Population‐based genetic testing for precision prevention. Cancer Prev Res. 2020;13(8):643‐648. doi:10.1158/1940-6207.CAPR-20-0002 32409595

[15] Katapodi MC , Duquette D , Yang JJ , et al. Recruiting families at risk for hereditary breast and ovarian cancer from a statewide cancer registry: a methodological study. Cancer Causes Control. 2017;28(3):191‐201. doi:10.1007/s10552-017-0858-2 28197806

[16] Hill DA , Haas JS , Wellman R , et al. Utilization of breast cancer screening with magnetic resonance imaging in community practice. J Gen Intern Med. 2018;33(3):275‐283. doi:10.1007/s11606-017-4224-6 29214373 PMC5834962

[17] Ozanne EM , Drohan B , Bosinoff P , et al. Which risk model to use? Clinical implications of the ACS MRI screening guidelines. Cancer Epidemiol Biomark Prev. 2013;22(1):146‐149. doi:10.1158/1055-9965.EPI-12-0570 23093547

[18] Silver E , Wenger N , Xie Z , et al. Implementing a population‐based breast cancer risk assessment program. Clin Breast Cancer. 2019;19(4):246‐253.e2. doi:10.1016/j.clbc.2019.02.009 31072694

[19] Bright Pink . Assess your risk. Accessed July 30, 2021.

[20] Welch BM , Allen CG , Ritchie JB , Morrison H , Hughes‐Halbert C , Schiffman JD . Using a chatbot to assess hereditary cancer risk. JCO Clin Cancer Inform. 2020;4:787‐793. doi:10.1200/CCI.20.00014 32897737 PMC7529541

[21] Daughter, Sister, Mother project at Padamsee lab. The JAMES—OSUCCC. Accessed February 28, 2022. https://cancer.osu.edu/for‐cancer‐researchers/research/research‐labs/padamsee‐lab/daughter‐sister‐mother‐project

[22] NIH RePORTER . Preventing breast cancer: decisions and effects among women at elevated risk. Project Number 5K01CA181547–05. Accessed February 22, 2022. https://reporter.nih.gov/search/lq1OVd682ESVC2tQ6c96GA/project‐details/9544050

[23] Padamsee TJ , Bijou C , Swinehart‐Hord P , Hils M , Muraveva A , Meadows RJ , Shane‐Carson K , Yee LD , Wills CE , Paskett ED . Risk management decision‐making data from a community‐based sample of racially diverse women at high risk of breast cancer: The *Daughter Sister Mother* Survey [Under Review]. 10.1186/s13058-023-01753-x PMC10785448 38212792

[24] Prince KR , Litovsky AR , Friedman‐Wheeler DG . Internet‐mediated research: beware of bots. Behav Ther. 2012;35(5):85‐88.

[25] Padamsee TJ , Meadows RJ , Kienzle S , Shane‐Carson K . Collecting non‐clinical data to address disparities in cancer prevention: lessons from the field [In preparation and available from authors.]

[26] StataCorp LLC . Stata Statistical Software. StataCorp LLC; 2019.

[27] Reid S , Cadiz S , Pal T . Disparities in genetic testing and care among black women with hereditary breast cancer. Curr Breast Cancer Rep. 2020;12(3):125‐131. doi:10.1007/s12609-020-00364-1 33603954 PMC7885902

[28] Williams CD , Bullard AJ , O'Leary M , Thomas R , Redding TS 4th , Goldstein K . Racial/ethnic disparities in BRCA counseling and testing: a narrative review. J Racial Ethn Health Disparities. 2019;6(3):570‐583. doi:10.1007/s40615-018-00556-7 30963508

[29] Randall TC , Armstrong K . Health care disparities in hereditary ovarian cancer: are we reaching the underserved population? Curr Treat Options in Oncol. 2016;17(8):39. doi:10.1007/s11864-016-0417-1 27315065

[30] Waters EA , Taber JM , McQueen A , Housten AJ , Studts JL , Scherer LD . Translating cancer risk prediction models into personalized cancer risk assessment tools: stumbling blocks and strategies for success. Cancer Epidemiol Biomark Prev. 2020;29(12):2389‐2394. doi:10.1158/1055-9965.EPI-20-0861 PMC8170537 33046450

[31] Kurian AW , Hughes E , Simmons T , et al. Performance of the IBIS/Tyrer‐Cuzick model of breast cancer risk by race and ethnicity in the Women's Health Initiative. Cancer. 2021;127(20):3742‐3750. doi:10.1002/cncr.33767 34228814

[32] Skinner CS , Ahn C , Singal AG , et al. Outcomes associated with use of the cancer risk intake system among primary care safety‐net patients identified as needing colorectal cancer screening. Prev Med Rep. 2019;16:101003. doi:10.1016/j.pmedr.2019.101003 31720201 PMC6838923

[33] Cleophat JE , Nabi H , Pelletier S , Bouchard K , Dorval M . What characterizes cancer family history collection tools? A Critical Literature Review. Curr Oncol. 2018;25(4):e335‐e350. doi:10.3747/co.25.4042 30111980 PMC6092046

