# Supplementary material for: Predicting breast cancer risk in a racially diverse, community‐based sample of potentially high‐risk women
Source: Cancer Med. 2022 Apr 6;11(21):4043–52. doi: 10.1002/cam4.4721 (PMC9636513; doi:10.1002/cam4.4721)
Supplement: Supplementary file 1 — Appendix S1 [file CAM4-11-4043-s001.docx]

**Supplemental Methods**

**Measures**

***Age.*** Age was collected through text-entry, then coded as a 5-level categorical variable: 18-24, 25-34, 35-44, 45-54, 55 and older.

***Race.*** Participants self-identified as either (1) White or (2) Black or African American. Other racial/ethnic categories were not eligible for this study.

***Education.*** Highest level of education was recoded into a 4-level categorical variable: (1) high school or GED, (2) some college or technical school, (3) college graduate or post-graduate, but no higher degree, and (4) post graduate degree.

***Annual household income.*** Annual household income (i.e., total pre-tax from all sources) was coded into five categories: (1) <$50,000, (2) $50,000-$99,000, (3) $100,000-$199,000 (4) ≥ $200,000 or (5) refused to answer or missing.

***Specialist care.*** Participants were asked to identify any healthcare providers they had ever seen including primary care providers, breast or cancer specialists, genetics specialists, women’s health specialists, and others.

**Risk Prediction Modeling**

Below we briefly describe the three risk prediction models on which this study is focused and the important differences between them; validation and characteristics of each model are discussed elsewhere.^1,2^

***Gail Model.***

The original Gail model estimates absolute risk (i.e., the probability of developing breast cancer over a defined age interval with allowance for competing risks) of invasive breast cancer up to 90 years of age. Therefore, the Gail model yields lower risk scores compared to other models that predict risk for both invasive and non-invasive cancers. The Gail model considers the number of maternal first-degree relatives with breast cancer including only mother, sisters, and daughters, but not paternal family history, age of first menstrual period, parity, age of first child, and results of breast biopsies (normal result or atypical hyperplasia). The Gail model has been validated in both White and Black/African American women and performs well in women ≥ 35 years of age who do not have pathogenic BRCA variants.^2^

***Claus Model.***

The original Claus model estimates cumulative risk of both invasive and non-invasive breast cancer up to age 80 years, based on age and family history alone. The model considers, and is intended for use on, women with at least one female first- or second-degree relative with breast cancer (i.e., mother, sisters, daughters, aunts, and grandmothers) on maternal and paternal sides.^3^

***IBIS (Tyrer-Cuzick) Model.***

The IBIS model (version 7) estimates cumulative risk of both invasive and non-invasive breast cancer up to age 85 years. IBIS considers family history of breast and ovarian cancer diagnoses, bilateral breast cancer, age of onset, and BRCA testing results for the following family members: parents, full siblings, half-sisters, children, aunts, cousins, nieces, and grandmothers on both maternal and paternal sides. IBIS also considers Ashkenazi inheritance, height and weight, parity and age of first child, and breast biopsy (normal result or atypical hyperplasia).^4^ The IBIS model has better predictive accuracy than other models when applied to high-risk populations based on family history.^5^

***Differences in Model Assumptions.***

The three models differ in their assumptions about the relationships between family history of cancer and personal cancer risk: the Gail model is based not a preconceived theory about hereditary patterns of breast cancer but on empirical incidence data; the Claus model assumes breast cancer is an autosomal dominant disease; the IBIS model allows for other genetic effects to account for residual familial correlation that is not explained only by autosomal dominance.^2^

**Supplemental Results and Discussion**

Supplemental analyses were conducted to investigate differences in the number of models run and resulting risk estimates by subgroups within the high-risk subsample. These subgroups are distinguished by sociodemographic factors, recruitment source, and whether participants had ever seen a specialist.

**Missing data**

There were no missing data for several main variables (e.g., age, race, family history). Income had the highest proportion of missing values (n=65, 9.1%). Participants with missing income were more likely to be White (OR=1.45 [95% CI: 0.82, 2.56]), either 18-24 years old (OR=5.34 [95% CI: 1.86, 15.39]) or 55 years or older (OR=5.99 [95% CI: 2.19, 16.41]), and have at least some college education (OR=1.85 [95% CI: 1.00, 3.43]).

**Group variation in number of risk models run**

***Results***

Supplementary Table 1 shows demographic differences in the number of models that could be run for subgroups of high-risk subsample participants. Because the Gail model requires age of 35 years or older, only participants aged 35 years and older were eligible to have all three risk models run. Compared to women ages 35-50 years, women ages 51-65 years had 35% (95% CI: 0.86, 2.13) higher odds of having 3 models run and women ages 66-74 years had 4.39 times (95% CI: 0.99, 19.45) the odds of having 3 models run. The number of models run was similar for White and Black/African American participants (OR=1.03 [95% CI: 0.80, 1.32]).

Participants with higher income were more likely to have all three models run. Each categorical increase in income level was associated with 18.4% (95% CI: 1.00, 1.40) higher odds of having all three models run compared to only one or two models. However, when controlling for age, the effect of income was attenuated (OR=0.98, [95% CI: 0.79, 1.21]).

Women recruited from Facing Hereditary Cancer Empowered (FORCE; a national nonprofit organization providing information and support to high-risk individuals) had 83% lower odds of having all three models run (OR=0.17, [95% CI: 0.094, 0.32]) compared to women recruited from ResearchMatch (a database of research volunteers), which may be explained by the fact that 96% of women recruited from FORCE reported pathogenic BRCA variants and therefore could not have the Gail model run. Lastly, the number of models run on each participant did not significantly differ by whether the participant had seen a breast or cancer specialist (OR=1.24 [95% CI: 0.92, 1.67]) or perceived risk (OR=1.02 [95% CI: 0.90, 1.15])

***Discussion***

Because the Gail model is only valid for women ages 35-85 and is inaccurate for women with pathogenic BRCA variants, this model was only run for 43% of high-risk subsample. Those who did not have the Gail model run were <35 years old (45%) or had known pathogenic BRCA variants (12.3%). The Claus model is not intended for use among women who have no first- or second- degree relatives with breast cancer, but such women can have other models run if they have third-degree relatives with breast or ovarian cancer. As a result of these factors, only 38% of the high-risk subsample could have all three risk prediction models run.

Older women and those with higher income were most likely to have all 3 models run (even among those age 35 and over, who are all eligible for the Gail model). When controlling for age, the associations with income were attenuated; this suggests that age may be a proxy for having access to more substantial family history information, which increases the chances that Claus (and therefore more total models) can be run.

Only a small proportion (8.8%) of our high-risk subsample were missing data that limited risk prediction modeling to only a single model (IBIS). This may be a result of our survey instrument, which was designed to collect all necessary information for risk prediction directly from participants. This represents a substantial improvement over other studies, which have reported insufficient information to run risk prediction models for 18-59% of women based on medical record data. Medical record data approaches likely result in disparities in risk prediction by race/ethnicity and socioeconomic status, since socioeconomically disadvantaged groups are more likely to have incomplete medical record data.^6^ For breast cancer risk management efforts to equitably reduce breast cancer burden, socioeconomically disadvantaged groups must be given equal opportunity for breast cancer risk prediction.

**Group variation in IBIS risk estimates**

***Results***

Since IBIS was run for all (except one) eligible participants, we used linear regression to explore the associations of IBIS risk estimates with sociodemographic and other characteristics (Supplementary Table 2). On average, women with pathogenic BRCA variants had 32.5% (95% CI: 0.31, 0.35) higher IBIS scores compared to women without such variants. Increasing age was associated with lower IBIS risk scores; this association was larger among pathogenic BRCA variant carriers than women without pathogenic BRCA variants (β= -0.44 [95% CI: -0.71, -0.16] vs β= -0.10 [95% CI: -0.14, -0.10], respectively). We observed no substantial differences in IBIS scores by race or income.

Those who reported having seen a cancer, breast, or genetics specialist had on average 2.5% (95% CI: 0.013, 0.037) higher IBIS scores than women who reported never having seen a specialist. Even among women without reported pathogenic BRCA variants, women recruited from FORCE had on average 7.4% (95% CI: 0.043, 0.11) higher IBIS scores than women recruited from ResearchMatch. Women recruited through a healthcare provider had on average 4.4% (95% CI: 0.017, 0.070) higher risk compared to women recruited from ResearchMatch.

***Discussion***

Overall, our results suggest that women with higher levels of health-seeking behavior may receive higher IBIS risk scores. Health-seeking behavior may motivate women to collect more substantial family history information, which can result in higher risk scores. Educational efforts about the importance of knowing and sharing family history of cancer and other breast cancer risk factors are crucial to maximizing equitable application of risk models in community and clinical settings.

**Group variations in risk discrepancies**

***Results***

Supplementary Table 3 presents results on factors associated with discrepancies in risk estimates between models among the high-risk subsample. Among women without reported BRCA variants who had 2 or 3 models run, discrepancies did not substantially differ by age, race, education, income, perceived risk, or having seen a specialist. For women without BRCA variants who had two models run, risk discrepancies varied by recruitment source: those recruited from FORCE had 7.7% (95% CI: 0.031, 0.12) larger risk discrepancies than women recruited from ResearchMatch. For women without pathogenic BRCA variants who had all three models run, however, there were no substantial differences by recruitment source.

Among women with pathogenic BRCA variants (who could only have two models run), we observed differences in the size of discrepancies by race, age, and having seen a specialist. White women had on average 15.1% (95% CI: -0.25, -0.053) larger discrepancies between risk estimates than Black/African American women. Smaller risk discrepancies were associated with increasing age, with women ages 66-74 years having on average 35% (95% CI: -0.78, -0.080) smaller discrepancies between risk estimates compared to women 18-24 years old. Women who had seen a specialist had on average 15% (95% CI: -0.0057, 0.31) larger risk discrepancies than women who had never seen a specialist.

***Discussion***

Larger discrepancies for younger women may result from differences in risk models accounting for older women “outliving” some of their risk compared to younger women with the same risk factors. Risk prediction models reduce cancer risk estimates by different amounts for the same increase in age, keeping other factors constant. In addition, women who have lived to later age without cancer are assumed less likely to share the genetic and environmental risk factors that may have caused cancer among their affected relatives. Younger women may therefore have a larger range of risk from various models, while older women have lower lifetime risk that corresponds to smaller discrepancies between models.

Larger discrepancies observed among women who have seen a specialist may be related to increases in risk estimates caused by health-seeking behaviors. Specialist care related to breast cancer risk and personal genetics may act as an educational intervention to prompt women to gather more full and accurate information about their family cancer history and other factors that are included in risk prediction models. The IBIS model incorporates a variety of additional factors (e.g., body mass index and hormone replacement therapy) that can result in higher calculated scores than other models; more thorough knowledge of personal breast cancer risk factors can also drive higher discrepancies between IBIS and other models.

White women had larger discrepancies than Black/African American women in this sample, which may partially be explained by the higher prevalence of pathogenic BRCA variants among White women compared to Black/African American women in this sample (26% vs. 11% respectively) since these variants are associated with larger discrepancies in risk scores. Discrepancies by race may also be related to differences in the cohorts used to develop the risk models. The IBIS model version 7, which drives higher discrepancies by giving the highest risk estimates compared to Gail and Claus, was originally validated in a general UK population cohort and may not be as applicable to Black/African Americans. Additionally, White women in the U.S. do have higher breast cancer incidence than Black/African American women (132.8 vs 121.2 per 100,000 persons respectively), which might not be captured to the same extent by all models and might help drive the higher discrepancy between models among White women.^7^ Improvement in risk prediction models, with inclusion of socioeconomically and racial/ethnically diverse women, may help minimize discrepancies between models and uncertainty in risk estimates.

**Supplemental References**

1. Cintolo-Gonzalez JA, Braun D, Blackford AL, et al. Breast cancer risk models: a comprehensive overview of existing models, validation, and clinical applications. *Breast Cancer Res Treat*. 2017;164(2):263-284. doi: 10.1007/s10549-017-4247-z.
2. Gail MH. Twenty-five Years of Breast Cancer Risk Models and Their Applications. *JNCI J Natl Cancer Inst*. 2015;107(5). doi:10.1093/jnci/djv042
3. Claus EB, Risch N, Thompson WD. Autosomal dominant inheritance of early-onset breast cancer. Implications for risk prediction. *Cancer*. 1994;73(3):643-651. doi: 10.1002/1097-0142(19940201)73:3<643::aid-cncr2820730323>3.0.co;2-5.
4. Tyrer J, Duffy SW, Cuzick J. A breast cancer prediction model incorporating familial and personal risk factors. *Stat Med*. 2004;23(7):1111-1130. doi:10.1002/sim.1668
5. Quante, AS, Whittemore, AS, Shriver, T, Strauch, K, & Terry, MB. (2012). Breast cancer risk assessment across the risk continuum: genetic and nongenetic risk factors contributing to differential model performance. *Breast cancer research*, *14*(6), R144. doi: 10.1186/bcr3352
6. Sholle, ET, Pinheiro, LC, Adekkanattu, P, Davila, MA, Johnson, SB, Pathak, J, Sinha, S, Li, C, Lubansky, SA, Safford, MM, & Campion, TR. (2019). Underserved populations with missing race ethnicity data differ significantly from those with structured race/ethnicity documentation. *Journal of the American Medical Informatics Association*, *26*(8-9), 722–729. doi: 10.1093/jamia/ocz040
7. National Cancer Institute. (2012). SEER Cancer Statistics Review 1975-2009. Accessed August 10, 2021. https://seer.cancer.gov/archive/csr/1975_2009_pops09/results_merged/sect_04_breast.pdf

| Supplementary Table 1  *Unadjusted odds of having 3 risk models run compared to 1-2 risk models run among the high-risk subsample* | | |
| --- | --- | --- |
|  | Odds ratio (95% CI) | *p* |
| Age |  |  |
| 18-24yrs | n/a^1^ |  |
| 25-34yrs | n/a^1^ |  |
| 35-50yrs | ref |  |
| 51-65yrs | 1.35 (0.86, 2.13) | .20 |
| 66-74yrs | 4.39 (0.99, 19.5) | .05 |
| Race |  |  |
| White | ref |  |
| Black/African American | 1.03 (0.80, 1.32) | .82 |
| Income | 1.18 (1.00, 1.40) | .05 |
| Education |  |  |
| ≤High school | ref |  |
| Some college/ technical school | 0.65 (0.30, 1.42) | .28 |
| College | 0.60 (0.28, 1.28) | .19 |
| Postgraduate | 1.01 (0.48, 2.13) | .98 |
| Perceived risk | 1.02 (0.90, 1.15) | .79 |
| Seen a cancer, breast, or genetics specialist | 1.24 (0.92, 1.67) | .17 |
| Recruitment source |  |  |
| ResearchMatch | ref |  |
| Facebook | 2.39 (1.35, 4.20) | < .01 |
| Healthcare provider | 1.38 (0.68, 2.79) | .37 |
| FORCE | 0.17 (0.09, 0.32) | < .001 |
| Advertisement | 1.15 (0.48, 2.73) | .75 |
| *Note.* ^1^Only participants 35 years and older were eligible to have all 3 models run | | |

| Supplementary Table 2  *Unadjusted linear regression for differences in IBIS scores by sociodemographic, clinical, and study characteristics among the high-risk subsample* | | | | |  |
| --- | --- | --- | --- | --- | --- |
|  | β (95% CI) | *p* | | | |
| Pathogenic BRCA variant^1^ | 0.325 | 0.31, 0.35 | | | |
|  | No known pathogenic BRCA variants | | Pathogenic BRCA variant carriers | | |
|  | β (95% CI) | *p* | β (95% CI) | *p* | |
| Age |  |  |  |  | |
| 18-24yrs | ref |  | ref |  | |
| 25-34yrs | -0.01 (-0.03, 0.01) | .34 | -0.06 (-0.19, 0.08) | .42 | |
| 35-50yrs | -0.01 (-0.03, 0.01) | .34 | -0.14 (-0.28, -0.12) | .03 | |
| 51-65yrs | -0.04 (-0.06, -0.02) | < .001 | -0.27 (-0.41, -0.14) | < .001 | |
| 66-74yrs | -0.10 (-0.14, -0.10) | < .001 | -0.44 (-0.71, -0.16) | < .01 | |
| Race |  |  |  |  | |
| White | ref |  | ref |  | |
| Black/African American | -0.01 (-0.02, 0.004) | .17 | -0.070 (-0.15, 0.01) | .10 | |
| Income | 0.005 (-0.002, 0.01) | .17 | 0.033 (-0.01, 0.07) | .09 | |
| Education |  |  |  |  | |
| ≤High school | ref |  | ref |  | |
| Some college/ technical school | -0.018 (-0.05, 0.01) | .25 | -0.071 (-0.27, 0.13) | .48 | |
| College | -0.016 (-0.04, 0.01) | .29 | -0.062 (-0.25, 0.12) | .51 | |
| Postgraduate | 0.0069 (-0.02, 0.04) | .06 | -0.033 (-0.22, 0.15) | .72 | |
| Perceived risk | 0.010 (0.005, 0.15) | < .001 | 0.0088 (-0.01, 0.30) | .42 | |
| Seen a specialist | 0.025 (0.01, 0.04) | < .001 | 0.079 (-0.03, 0.19) | .14 | |
| Recruitment source |  |  |  |  | |
| ResearchMatch | ref |  | ref |  | |
| Facebook | 0.01 (-0.01, 0.03) | .51 | -0.02 (-0.21, 0.16) | .82 | |
| Healthcare provider | 0.04 (0.17, 0.07) | < .01 | -0.05 (0.23, 0.14) | .61 | |
| FORCE | 0.07 (0.04, 0.11) | < .001 | 0.04 (-0.06, 0.15) | .39 | |
| Advertisement | -0.02 (-0.06, 0.01) | .21 | n/a^2^ |  | |
| *Note.*  ^1^Among all 717 women who had IBIS run ^2^No BRCA variant carriers were recruited by physical advertisements | | | | |  |

| Supplementary Table 3  *Unadjusted linear regression for discrepancies in risk estimates between models sociodemographic, clinical, and study characteristics among the high-risk subsample* | | | | | | |
| --- | --- | --- | --- | --- | --- | --- |
| No known pathogenic BRCA variants  (3 models run, n=276) | | No known pathogenic BRCA  (2 models run, n=259) | | | Pathogenic BRCA variant carriers  (2 models run, n=113) | |
|  | β (95% CI) | *p* | β (95% CI) | *p* | β (95% CI) | *p* |
| Age |  |  |  |  |  |  |
| 18-24yrs | n/a^1^ |  | ref |  | ref |  |
| 25-34yrs | n/a^1^ |  | -0.022 (-0.04, -0.005) | .01 | -0.11 (-0.29, 0.08) | .26 |
| 35-50yrs | ref |  | -0.015 (-0.04, 0.01) | .29 | -0.17 (-0.35, 0.02) | .08 |
| 51-65yrs | -0.02 (-0.04, -0.10) | < .01 | -0.072 (-0.16, 0.02) | .10 | -0.26 (-0.45, -0.07) | .01 |
| 66-74yrs | -0.01 (-0.03, 0.02) | .66 | n/a^2^ |  | -0.35 (-0.78, 0.08) | .11 |
| Race |  |  |  |  |  |  |
| White | ref |  | ref |  | ref |  |
| Black/African American | 0.021 (0.01, 0.03) | < .01 | -0.0096 (-0.03, 0.01) | .22 | -0.15 (-0.25, -0.05) | < .01 |
| Income | -0.002 (-0.01, 0.005) | .56 | 0.00074 (-0.01, 0.01) | .86 | 0.015 (-0.03, 0.06) | .53 |
| Education |  |  |  |  |  |  |
| ≤High school | ref |  | ref |  | ref |  |
| Some college/ technical school | 0.010 (-0.02, 0.04) | .51 | -0.026 (-0.07, 0.01) | .20 | -0.00033 (-0.21, 0.21) | 1.00 |
| College | 0.0083 (-0.02, 0.04) | .59 | -0.035, (-0.07, 0.003) | .07 | 0.011 (-0.18, 0.21) | .91 |
| Postgraduate | 0.020 (-0.01, 0.049) | .19 | -0.021 (-0.06, 0.02) | .29 | 0.043 (-0.15, 0.24) | .66 |
| Perceived risk | 0.00053 (-0.005, 0.01) | .85 | 0.00077 (-0.01, 0.01) | .83 | -0.00037 (-0.03, 0.03) | .97 |
| Seen a specialist | 0.0066 (0.006, 0.19) | .30 | 0.0058 (-0.01, 0.02) | .54 | 0.15 (-0.01, 0.31) | .06 |
| Recruitment source |  |  |  |  |  |  |
| ResearchMatch | ref |  | ref |  | ref |  |
| Facebook | -0.024 (-0.04, -0.01) | .01 | -0.021 (-0.05, 0.01) | .19 | -0.056 (-0.27, 0.16) | .61 |
| Healthcare provider | 0.0073 (-0.02, 0.03) | .59 | -0.032 (-0.07, 0.01) | .11 | -0.13 (-0.36, 0.11) | .29 |
| FORCE | -0.014 (-0.04, 0.02) | .36 | 0.077 (0.03, 0.12) | < .01 | 0.058 (-0.07, 0.18) | .36 |
| Advertisement^3^ | -0.0032 (-0.06, 0.05) | .91 | -0.024 (-0.07, 0.02) | .27 | n/a^1^ |  |
| *Note.* ^1^Only participants 35 years and older were eligible to have all 3 models run ^2^No BRCA variant carriers in this age group who had 2 models run ^3^No BRCA variant carriers were recruited by physical advertisements | | | | | | |
